# Association Between Ambient Air Pollutants Exposure and Preterm Birth in Women Who Underwent *in vitro* Fertilization: A Retrospective Cohort Study From Hangzhou, China

**DOI:** 10.3389/fmed.2021.785600

**Published:** 2021-12-13

**Authors:** Wenming Shi, Meiyan Jiang, Lena Kan, Tiantian Zhang, Qiong Yu, Zexuan Wu, Shuya Xue, Xiaoyang Fei, Changbo Jin

**Affiliations:** ^1^Shanghai First Maternity and Infant Hospital, Tongji University School of Medicine, Shanghai, China; ^2^School of Public Health, Li Ka Shing Faculty of Medicine, The University of Hong Kong, Hong Kong, Hong Kong SAR, China; ^3^Department of Reproductive Medicine, Hangzhou Women's Hospital, Hangzhou, China; ^4^Division of Population, Family and Reproductive Health, Bloomberg School of Public Health, Johns Hopkins University, Baltimore, MD, United States; ^5^School of Public Health, Fudan University, Shanghai, China; ^6^Reproductive Medicine Center, Tongji Hospital, Tongji Medical College, Huazhong University of Science and Technology, Wuhan, China; ^7^Department of Reproductive Center, Peking University Shenzhen Hospital, Shenzhen, China; ^8^Hangzhou Medical College, Hangzhou, China; ^9^Shanghai Key Laboratory of Maternal-Fetal Medicine, Shanghai First Maternity and Infant Hospital, Tongji University School of Medicine, Shanghai, China

**Keywords:** air pollution, preterm birth, assisted reproductive technology, PM_2.5_, nitrogen dioxide

## Abstract

**Objectives:** Exposure to air pollutants has been linked to preterm birth (PTB) after natural conception. However, few studies have explored the effects of air pollution on PTB in patients who underwent *in vitro* fertilization (IVF). We aimed to investigate the association between ambient air pollutants exposure and PTB risk in IVF patients.

**Methods:** This retrospective cohort study included 2,195 infertile women who underwent IVF treatment from January 2017 and September 2020 in Hangzhou Women's Hospital. Totally 1,005 subjects who underwent a first fresh embryo(s) transfer cycle were analyzed in this study. Residential exposure to ambient six air pollutants (PM_2.5_, PM_10_, SO_2_, NO_2_, CO, O_3_) during various periods of the IVF timeline were estimated by satellite remote-sensing and ground measurement. Cox proportional hazards models for discrete time were used to explore the association between pollutants exposure and incident PTB, with adjustment for confounders. Stratified analyses were employed to explore the effect modifiers.

**Results:** The clinical pregnancy and PTB rates were 61.2 and 9.3%, respectively. We found that PM_2.5_ exposure was significantly associated with an increased risk of PTB during 85 days before oocyte retrieval [period A, adjusted hazard ratio, HR=1.09, 95%CI: 1.02–1.21], gonadotropin start to oocyte retrieval [period B, 1.07 (1.01–1.19)], first trimester of pregnancy [period F, 1.06 (1.01–1.14)], and the entire IVF pregnancy [period I, 1.07 (1.01–1.14)], respectively. An interquartile range increment in PM_10_ during periods A and B was significantly associated with PTB at 1.15 (1.04–1.36), 1.12 (1.03–1.28), and 1.14 (1.01–1.32) for NO_2_ during period A. The stratified analysis showed that the associations were stronger for women aged <35 years and those who underwent two embryos transferred.

**Conclusions:** Our study suggests ambient PM_2.5_, PM_10_, and NO_2_ exposure were significantly associated with elevated PTB risk in IVF patients, especially at early stages of IVF cycle and during pregnancy.

## Introduction

Preterm birth (PTB, <37 weeks of gestation) has become the leading cause of infant mortality and neonatal morbidity (1). It has been estimated that about 2.7 million (1.8–3.5 million) births globally were preterm in 2010 (2), while the rate of PTB in China was 7.1% in the same year, ranking second after India (3). Studies have indicated that PTB may not only lead to direct death and disability in neonates, but also accounts for increased risks of respiratory (4), cardiovascular (5), and metabolic diseases (6, 7)in later life. Acknowledging the heavy burden that PTB imposes on both infants and the wider society, identification of its risk factors is imperative.

In recent years, a growing number of studies have focused on the effects of air pollutants on adverse pregnancy outcomes, including PTB (8–11). As the largest developing country in the world, China is now facing severe air pollution-related challenges. Several studies have demonstrated that air pollutants exposure may be associated with an increased risk of PTB (2, 8, 12, 13). However, the results were still inconsistent, and the mechanisms whereby air pollutants could increase the PTB risk remain inconclusive. Oxidative stress, endothelial dysfunction, respiratory infections, hemodynamic responses, and endocrine disruption are of primary interest to determine whether any of these effects are attributed with PTB (14). Identifying the susceptible exposure windows would be helpful to illuminate the possible mechanism of air pollutants on adverse pregnancy outcomes. However, most previous studies were conducted among pregnancies achieved through natural conception, and although specific trimesters during pregnancy were examined, the effects of air pollution during the period before pregnancy remain unclear.

Since 1978, more than five million children were born through assisted reproductive technology (ART) treatment (15). This provides the opportunity for a novel evaluation of the effects of air pollutants because it is possible to observe many early reproductive outcomes, such as embryo implantation that can't be observed in natural pregnancy (16). Furthermore, the timing of conception is known with certainty because key events in the IVF treatment are timed and trigger by a physician, exact exposure periods of air pollutants can be determined (16, 17). To date, several studies have reported the association between air pollutants exposure and follicle growth, a reduced rate of implantation, pregnancy or live birth in women who underwent ART treatment, but the results were inconsistent (16, 18–21). However, few epidemiology studies have explored the effects of ambient air pollution during different time windows on PTB incidence in patients who underwent IVF treatment in China.

Therefore, we addressed this gap in knowledge by investigating the association between ambient air pollutants exposure and the incident risk of PTB in women who underwent IVF treatment in Hangzhou, China, in a retrospective cohort study. Furthermore, we explored the potential time windows of exposure to air pollutants that could serve as a reference for identifying the biological mechanisms of PTBs and thereby decreasing the overall disease burden attributed to air pollution.

## Methods

### Study Design and Participants

**Figure 1:**
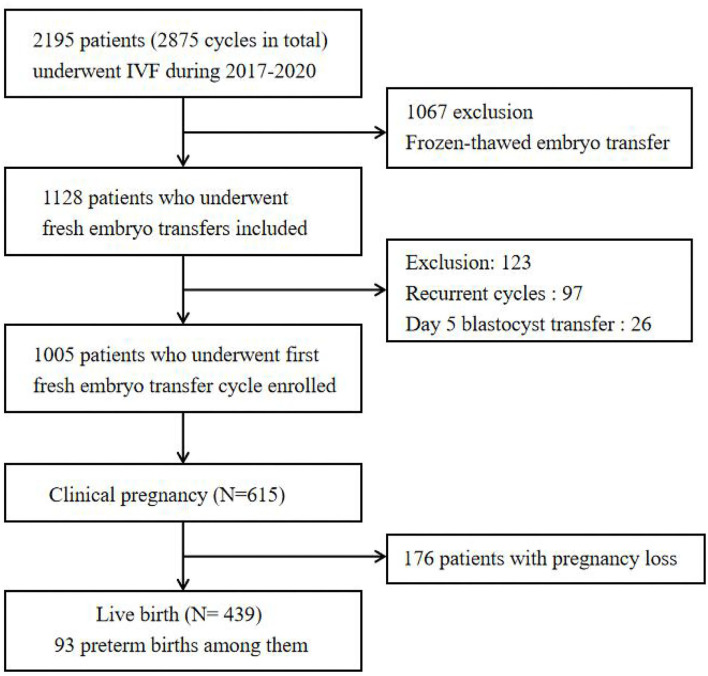
The flowchart of the participants enrollment.

We performed a retrospective cohort study among 2,195 patients, aged 20 to 43 years, living in Hangzhou, who underwent IVF cycles at the Department of Reproductive Medicine of Hangzhou Women's Hospital (latitude 30°23′ N, longitude 120°20′ E) between January 1, 2017 and September 30, 2020. During the entire IVF cycle, no subjects reported relocating or changing their residential address. Participants were excluded if they (1) underwent frozen–thawed embryos transfer, (2) received day 5 blastocyst transfer, or (3) underwent recurrent embryo transfer cycles. Finally, 1,005 patients who underwent a first, fresh embryo transfer in first cycle were enrolled in our study. The flowchart of the participants included in this study is shown in Figure 1. All data were anonymous and stripped of any identifier.

### IVF Procedures

The integrated IVF procedure included four stages: controlled ovarian stimulation (COS), oocyte retrieval, embryo transfer, and pregnancy test. The best ovulation scheme and the initial dose of exogenous gonadotropins (Gn) were determined according to the patient's age, body mass index (BMI), and ovarian reserve. Generally, participants underwent one of three COS protocols according to their ovarian reserve: the long gonadotropin-releasing hormone (GnRH)-agonist (-a) regimen (generally used for normal/high responders), GnRH antagonist (-ANT) regimen (generally used for high responders), and other regimens (such as mild stimulation regimens and progesterone-activated ovarian stimulation regimens [PPOS]). In the GnRH-ANT regimen, subjects were injected daily with recombinant or human menopausal gonadotropin (Gonal-F or Puregon or HMG) from day 2 or day 3 of the menstrual cycle. When at least one follicle reached 12 mm in size or after stimulating to day 6, a gonadotropin-releasing hormone antagonist, 0.125 mg or 0.25 mg, was injected subcutaneously daily until the trigger day. The long GnRH-a regimen started a with GnRH agonist (subcutaneous injection of triptorelin 0.1 mg/d) 7–10 days before the menstrual cycle, followed by gonadotropin therapy 14 days later. In the mild stimulation regimen or the PPOS regimen, subjects were given clomiphene citrate (50 or 100 mg) or a progesterone capsule (200 g) on the second or third day of the menstrual cycle, respectively, and gonadotropin was administered simultaneously. When the diameter of two or more follicles reached ≥18 mm, 10,000 U hCG (Lizhu Pharmaceutical Co., Ltd. China) was injected subcutaneously on the trigger day. For subjects with a high response >20 follicles, 4,000–5,000 IU hCG was injected subcutaneously on the trigger day. About 36–37 h after the hCG trigger, eggs were collected using transvaginal ultrasound. Semen was collected through masturbation, and spermatozoa were prepared by density gradient centrifugation. Embryologists chose IVF and/or intracytoplasmic sperm injection (ICSI) according to sperm quality. If the 2PN embryo had 7–10 blastomeres, no nuclear fragments <5%, and no obvious morphological abnormalities, the embryo was considered of good quality by the third day. For patients who received fresh embryo transfer, one or two embryos were transferred on the third day after egg extraction, under the guidance of transabdominal ultrasound. Luteal support began on the day of egg collection. The remaining embryos were frozen according to the standard procedure of the Center. Among women who tested positive for hCG on the 14th day after transplantation, luteal phase support lasted until 10 weeks of pregnancy. Clinical pregnancy was defined when an intrauterine pregnancy sac was identified by ultrasound 5 weeks (35 days) after embryo transfer and abortion was defined as spontaneous abortion before 28 weeks. Live birth was defined as all live births in embryo transfer cycles, and PTB was defined as babies born at <37 weeks of gestational age.

### Ambient Air Pollutants Exposure

Ambient daily levels of fine particulate matter (PM_2.5_), inhalable particulate matter (PM_10_) were obtained from the China High Air Pollutants (CHAP) datasets. The detailed methodology was described previously (22–24). Briefly, it is generated from a combination of satellite- derived MODIS/Terra+Aqua MAIAC aerosol optical depth (AOD), ground-based monitoring, atmospheric reanalysis, and model stimulation. The randomized tree approach estimated the ambient concentration of PM_2.5_ and PM_10_ at ~1 ×1 km resolution with 10-fold cross-validation R^2^ of 0.87 and 0.73, respectively. In this study, geocoding was performed at each residential location of participants to estimate the exposure concentration of PM_2.5_ and PM_10_, and then calculated the average level during each time period.

For other criteria pollutants, ambient daily average of sulfur dioxide (SO_2_), nitrogen dioxide (NO_2_), carbon monoxide (CO) and ozone (O_3_) were measured from the nearest air monitoring station of each residential address, and obtained from the China National Environmental Monitoring Center (http://www.cnemc.cn) during the study period. The distance from each participant's residence to their nearest air monitoring station ranged from 0.11 to 9.97 km (mean: 3.24 km, median: 3.06 km). Available data of the station-specific air pollutants were spatially interpolated using inverse distance-squared weighting to estimate the exposure level of each participant during the study period (25, 26).

**Figure 2:**
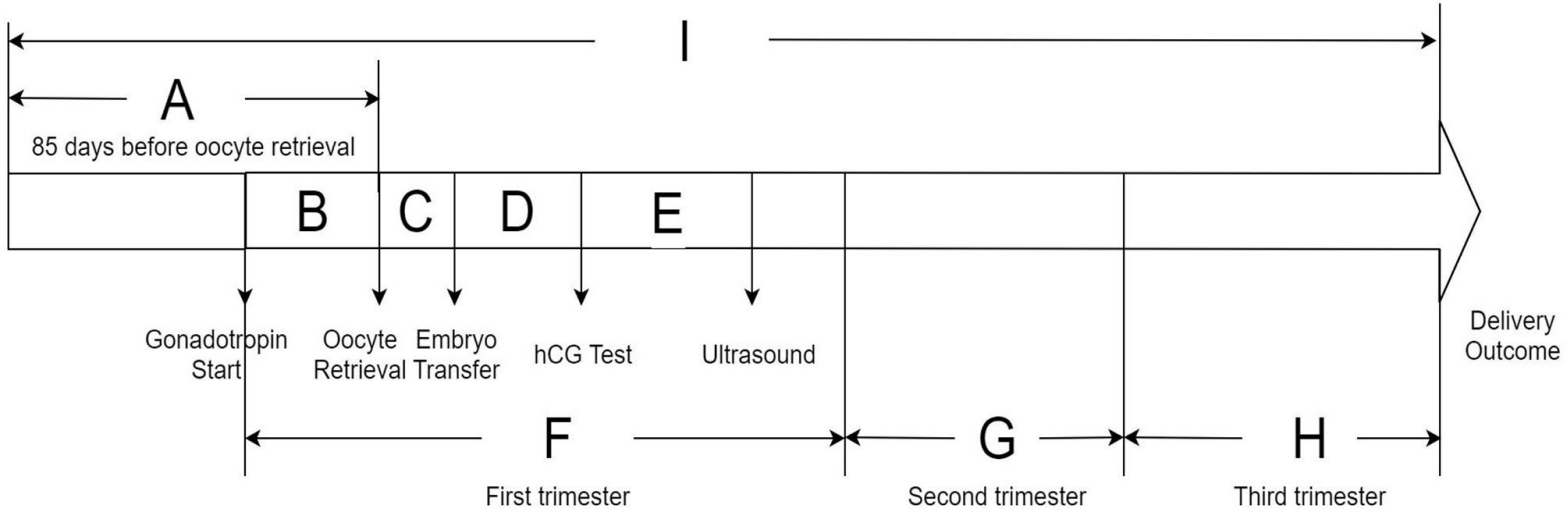
Timeline of exposure windows during a fresh *in vitro* fertilization cycle.

To evaluate the potential susceptible time windows, the study period for each subject was divided into nine discrete time periods based on the IVF timeline: (1) period A: 85 days before oocyte retrieval; (2) period B: from commencing gonadotropin treatment to oocyte retrieval; (3) period C: from oocyte retrieval to embryo transfer; (4) period D: from embryo transfer to serum hCG test; (5) period E: from serum hCG test to ultrasound test; (6) period F: the first trimester of pregnancy (from commencing gonadotropin treatment to gestational week 13 in IVF women); (7) period G: the second trimester of pregnancy (from gestational week 14–26); (8) period H: the third trimester of pregnancy (from gestational week 27 to delivery outcome); (9) period I: from 85 days before oocyte retrieval to delivery outcome. The timeline of the research periods of the whole IVF pregnancy is presented in Figure 2. We calculated the average exposure concentration of each air pollutant during different periods for each participant.

### Covariates

At enrollment, information on maternal age, maternal pre-pregnancy body mass index (BMI), educational level, employment status, residential locations (urban vs. non-urban, which defined by the Chinese administrative division), and cigarette smoking exposure (yes or no) were collected from medical records. Education level was classified into three groups: ≤ middle school; high school, and ≥ junior college school. In addition, the covariables related to IVF treatment, including the duration of infertility, infertility diagnosis (which were classified into three groups: male factor; female factor, and other factors), oocyte insemination technique, treatment protocols, number of oocytes retrieved, normal (2PN) fertilized oocytes, number of embryos transferred (one or two) and year of embryo transfer were also recorded. Daily ambient temperature and relative humidity (RH) were derived from China Meteorological Data Service Center (http://data.cma.cn/en) and averaged for each time period.

### Statistical Analyses

Descriptive statistics were performed for all clinical and environmental data. The continuous variables are expressed as mean ± standard deviations (SD) and the categorical variables are summarized as counts and percentages. Spearman's rank correlation analysis was used to evaluate the correlations across air pollutants as well as within pollutants over time. A potential collinearity was considered when the Spearman's correlation coefficients of pairwise pollutants were higher than 0.60.

Multivariable Cox proportional hazards models for discrete time periods were used to explore the associations between ambient air pollutants and PTB incidence in IVF patients. Compared to the “odds ratio” from logistic regression model, Cox model provide a more straightforward interpretation that translates the hazard ratio into “risk”. Potential confounders were selected from known or potential PTB influencing factors based on current knowledge. Multivariable adjusted models included the following covariates: maternal age, maternal pre-pregnancy BMI, educational level, employment status, cigarette smoking exposure, residential locations, ambient average temperature and RH. Results from the models were calculated as the adjusted hazard ratios (HR) and 95% confidence intervals (CIs) of PTB for each interquartile range (IQR) increment in ambient air pollutants during each period.

To explore the potential effect modifications, we fitted separate models and obtained the HRs and 95%CIs by following factors: maternal age (which was classified into two subgroups: ≥ 35 years or <35 years), oocyte insemination technique (IVF vs. ICSI), and treatment protocol (long GnRH agonist vs. GnRH antagonist), and number of embryo(s) transferred (one embryo vs. two embryos), respectively.

In addition to main analyses, we performed two sensitivity analyses. Firstly, we examined the robustness of the associations by conducting a two-pollutant model to mutually adjusted the potential confounding from the co-pollutants. Secondly, we ran the multivariate regression model by further controlling for the average concentration of air pollutants during the baseline period (period A) to evaluate the independent effects of short-term variations in air pollutants.

All statistical analyses were performed by using STATA 16.0 (Stata Corporation, College Station). All *p* values were two sided, and statistical significance was defined as *p* < 0.05.

## Results

In this study, we included 1,005 patients who underwent a first, fresh embryo transfer in the first cycle in Hangzhou, China. The average age and BMI of the subjects were 31.78 ± 4.02 years and 22.17 ± 3.17 kg/m^2^, respectively. The clinical pregnancy, live birth, and PTB rate among the study participants were 61.2, 43.7, and 9.3%, respectively. Detailed statistics of the demographics and clinical characteristics of the participants are shown in Table 1.

**Table 1:** Descriptive statistics regarding subject demographic and clinical characteristics.

**Variables**	**Outcome of fresh IVF cycle**		
	**Entire cohort**	**Clinical pregnancy^**a**^**	**PTB^**a**^**
	**(*N* = 1,005)**	**(*N* = 615)**	**(*N* = 93)**
**Maternal age (years)**	31.78 ± 4.02	31.27 ± 3.79	31.43 ± 325
**Maternal pre-pregnancy BMI (kg/m** ^**2**^ **)**	22.17 ± 3.17	22.25 ± 3.19	21.71 ± 3.26
**Educational level (%)**			
≤ Middle school	211 (21.0)	134 (63.5)	21 (10.0)
High school	341 (33.9)	223 (65.4)	33 (9.7)
≥ Junior college school	453 (45.1)	258 (57.0)	39 (8.6)
**Employment status (%)**			
Currently working	909 (90.5)	566 (62.3)	84 (9.2)
Currently not working	96 (9.5)	49 (51.0)	9 (9.4)
**Cigarette smoking exposure (%)**			
Yes	19 (1.9)	11 (57.9)	2 (10.5)
No	986 (98.1)	604 (61.3)	88 (8.9)
**Residential location (%)**			
Urban	912 (90.7)	556 (61.0)	85 (9.3)
Non-urban	93 (9.3)	59 (63.4)	8 (8.6)
**IVF cycle characteristics**			
**Duration of infertility (years)**	3.37 ± 2.44	3.34 ± 2.33	3.57 ± 2.31
**Infertility diagnosis (%)**			
Male factor	112 (11.1)	67 (59.8)	14 (12.5)
Female factor	732 (72.9)	442 (60.4)	63 (8.6)
Unexplained	161 (16.0)	106 (65.8)	16 (9.9)
**Treatment protocol (%)**			
Long GnRH agonist	806 (80.2)	502 (62.3)	77 (9.6)
GnRH antagonist	150 (14.9)	86 (57.3)	14 (9.3)
Others	49 (4.9)	27 (55.1)	2 (4.1)
**Oocyte insemination technique (%)**			
IVF	870 (86.6)	543 (62.4)	80 (9.2)
ICSI	135 (13.4)	72 (53.3)	13 (9.6)
**Number of oocytes retrieved**	8.85 ± 3.71	8.88 ± 3.71	8.62 ± 3.89
**Normal (2PN) fertilized oocytes**	5.90 ± 3.03	6.00 ± 3.05	6.05 ± 3.33
**Number of embryos transferred (%)**			
One embryo	156 (15.5)	69 (44.2)	5 (3.2)
Two embryos	849 (84.5)	546 (64.3)	88 (10.3)
**Year of embryo transfer (%)**			
2017	254 (25.3)	154 (60.6)	24 (9.4)
2018	269 (26.8)	164 (61.0)	25 (9.3)
2019	250 (24.9)	155 (62.0)	23 (9.2)
2020	232 (23.1)	142 (61.2)	21 (9.1)
**Meteorological factors**			
Ambient temperature (°C)	18.1 ± 3.2	18.3 ± 3.7	18.5 ± 4.0
Ambient RH (%)	65.9 ± 7.1	66.2 ± 7.0	66.5 ± 7.7

*Data are given as mean ± standard deviation or N (%).*

a *, Indicates the number and rate of the pregnancy or preterm birth.*

*IVF, in vitro fertilization; ICSI, intracytoplasmic sperm injection; PTB, preterm birth; BMI, body mass index; GnRH, gonadotropin-releasing hormone; RH, Relative humidity*.

The ambient average levels of six air pollutants during the different time periods are presented in Supplementary Figure S1. The mean (range) level of ambient PM_2.5_ exposure was 36.02 (23.3–47.1) μg/m^3^; PM_10_, 64.13 (39.78–104.44) μg/m^3^; SO_2_, 8.02 (5.13–10.93) μg/m^3^; NO_2_, 38.35 (22.77–51.75) μg/m^3^; CO, 0.82 (0.69–0.92) mg/m^3^, O_3_, 56.42 (45.45–76.36) μg/m^3^ during the whole IVF pregnancy among the subjects (Supplementary Figure S1). Correlation analyses showed that air pollutants were moderately to highly correlated with each other during each exposure period. Ozone was negatively correlated with other air pollutants (Supplementary Table S1). Across the timeline of the IVF cycle, specific air pollutants were moderately to highly correlated. The details about the correlation coefficient are clearly presented in Supplementary Table S2.

**Figure 3:**
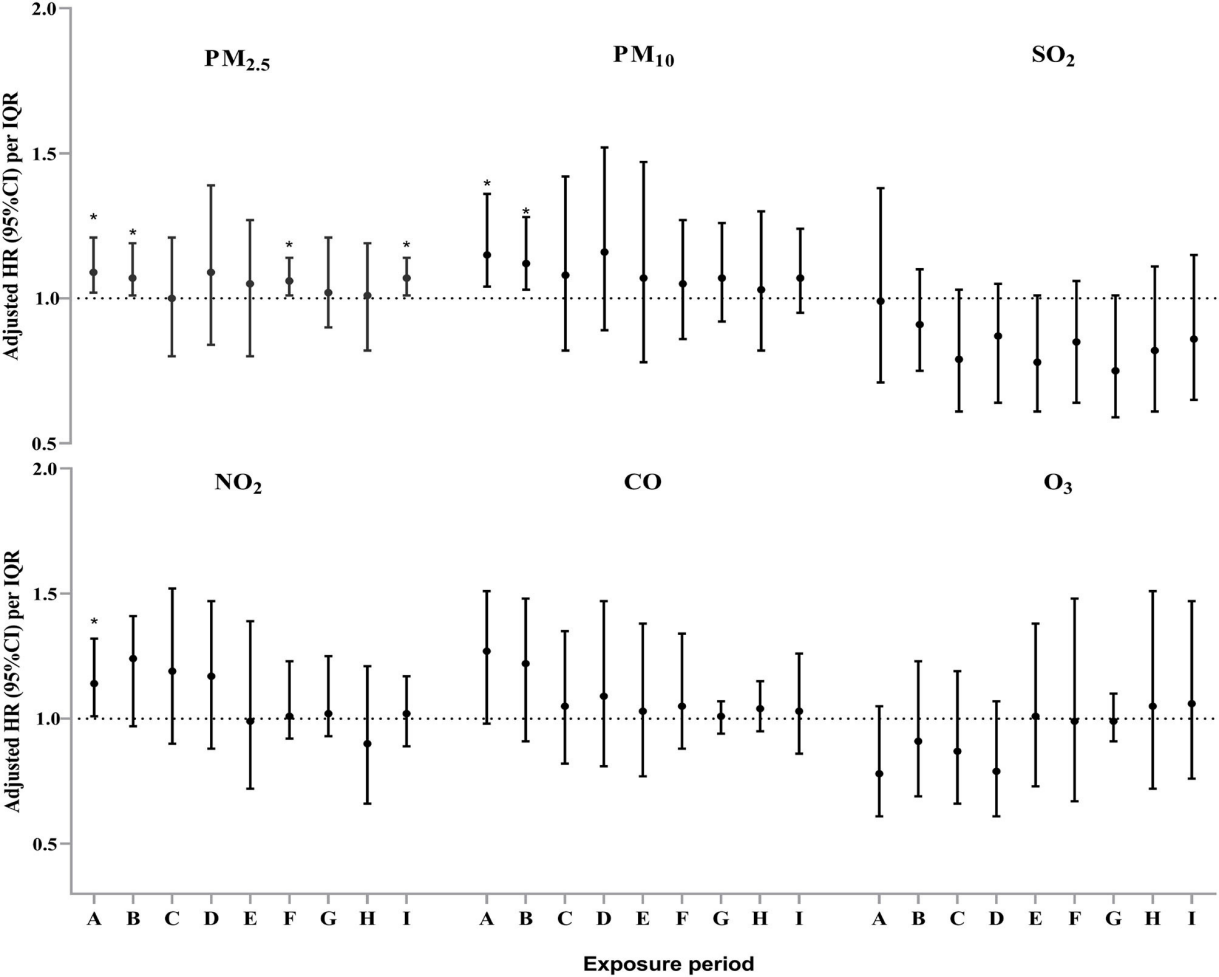
Adjusted hazard ratio (HR) and 95% confidence interval (CI) for the association between exposure to six air pollutants and the incident risk of preterm birth during different exposure periods among the study subjects^#^. ^#^ Model adjusted for maternal age, maternal pre-pregnancy BMI, educational level, employment status, cigarette smoking exposure, residential locations, ambient average temperature, and relative humidity. *P < 0.05.

The multivariable adjusted Cox proportional hazards modeling showed that ambient PM_2.5_ exposure was significantly associated with an increased risk of PTB incidence among the participants during period A (85 days before oocyte retrieval), period B (gonadotropin start to oocyte retrieval), period F (the first trimester of pregnancy) and period I (from 85 days before oocyte retrieval to delivery outcome). The adjusted HR and 95%CIs were 1.09 (1.02–1.21), 1.07 (1.01–1.19), 1.06 (1.01–1.14) and 1.07 (1.01–1.14) for each IQR increment in PM_2.5_ exposure levels during the aforementioned periods, respectively (Figure 3). After further adjusting for other co-pollutants in the regression model, except for CO, robust associations were observed for ambient PM_2.5_ exposure levels in the two-pollutant models during the whole IVF pregnancy (Supplementary Table S3). For PM_10_, we found that each IQR increment in ambient PM_10_ exposure concentration was significantly associated with an increased PTB risk during period A and period B (Figure 3). The adjusted HRs and 95% CIs were 1.15 (1.04–1.36) and 1.12 (1.03–1.28), respectively. Furthermore, a significant association between ambient nitrogen dioxide (NO_2_) exposure and incident PTB during period A was observed (HR=1.14, 95% CI: 1.01–1.32) (Figure 3). However, no significant associations of PTB risk with ambient SO_2_, CO, and O_3_ were identified in this study.

**Figure 4:**
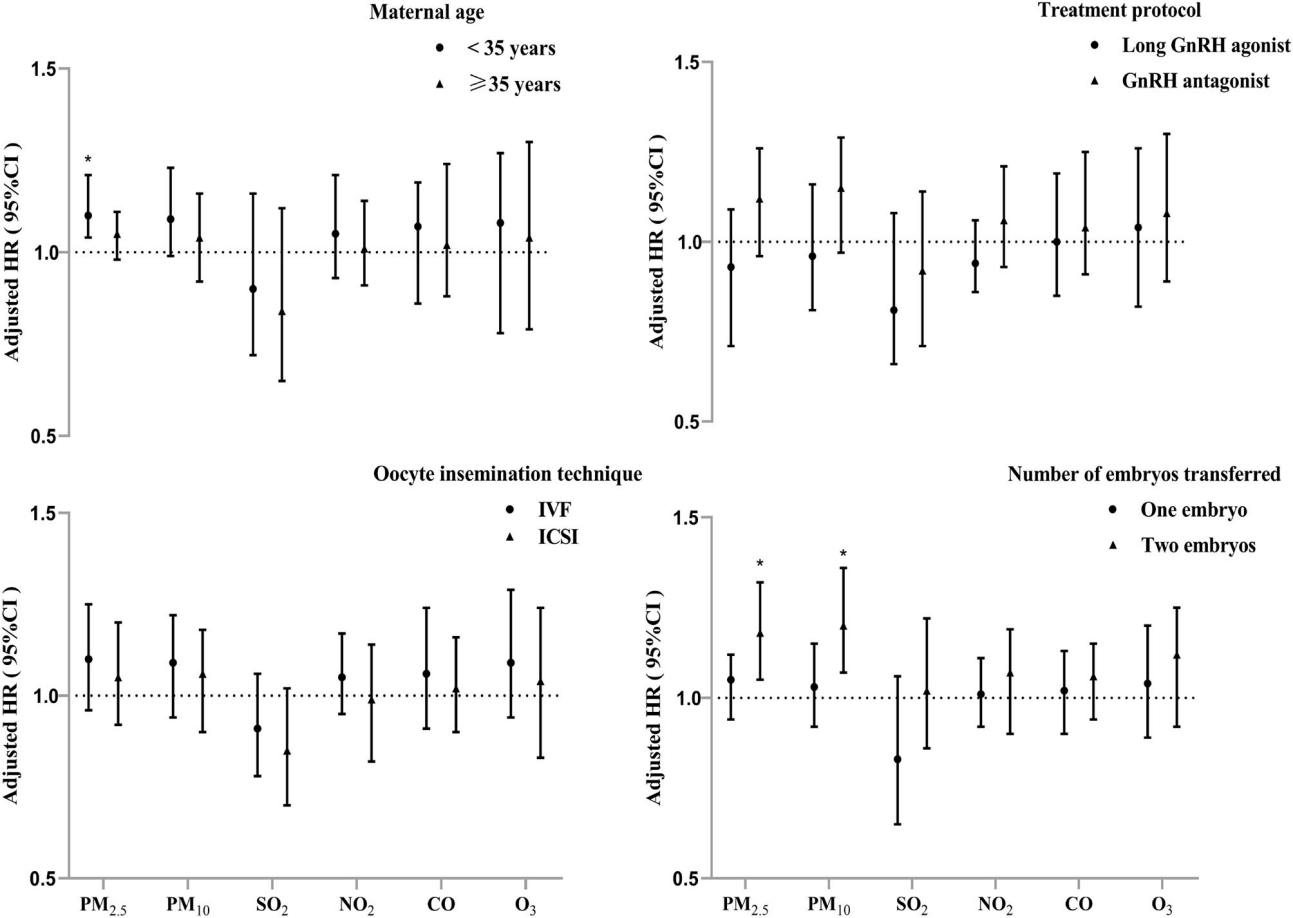
Adjusted hazard ratios (95% confidence intervals) of PTB risk per interquartile range increment in air pollutants exposure during IVF pregnancy (period I) stratified by potential modifiers. *P < 0.05.

We found stronger associations of ambient particulate matter exposure during the entire IVF pregnancy (period I) with PTB risk among patients who aged <35 years, and those who underwent two embryos transferred (Figure 4). The stratified analyses showed that each IQR increment in PM_2.5_ concentration during period I was significantly associated with increase of PTB risk by 10% among women who aged <35 years. Similarly, a higher risk of PTB associated with ambient PM_2.5_ and PM_10_ was observed among the subjects who underwent two embryos transferred than those had single embryo transferred (Figure 4). However, no significant effect modification was found for treatment protocols and oocyte insemination technique among the subjects.

The sensitivity analyses showed no significant changes on the associations for time-varying PM_2.5_ and PM_10_ exposure during each period and the PM further adjusted for the baseline exposure level during the 85 days before oocyte retrieval (Supplementary Figure S2).

## Discussion

This retrospective cohort study investigated the association between ambient air pollutants exposure during different time windows and the incident risk of PTB in patients who underwent IVF treatment in China. We observed significant associations between time-varying PM_2.5_, PM_10_, and NO_2_ exposure and the increased PTB risks of infertility treatment with IVF. The specific adverse effects of these air pollutants during the exposure periods 85 days before oocyte retrieval, from gonadotropin start to oocyte retrieval, first trimester of pregnancy, and from 85 days before oocyte retrieval to delivery outcome on PTB incidence were identified. The detrimental effects were more pronounced in younger women (aged <35 years) and those who undergoing two embryos transferred. However, no significant associations were observed for PTB outcome with SO_2_, CO, and O_3_ in this study.

Similar to a previous study in IVF patients (18), the clinical pregnancy rate in our study was 61.2%. A study performed in Shengyang, China showed that the intrauterine pregnancy rate was 46.0% (27), which was lower than the recorded rate in our study. In terms of PTB rate, our finding (9.3%) was slightly higher than those reported in two prior studies involving natural conception (8.0 and 8.3%, respectively) (28, 29), indicating that IVF patients might have a higher risk of PTB. A reason for this might be that women who undergo IVF are more likely to have multiple pregnancies than those who conceive naturally, because multiple embryos might be transferred during treatment. Multiple pregnancies can pose increased risks for both mothers and for infants, such as PTB and low birth weight (30).

The average exposure concentrations of ambient PM_2.5_ during the entire study period were around 36.0 μg/m^3^, which is three times greater than what is recommended by the WHO Air Quality Guidelines (10 μg/m^3^ of PM_2.5_). In the current study, we explored the effects of ambient PM_2.5_ exposure during nine different time periods throughout the IVF cycle on the risk of PTB and observed significant associations for PM_2.5_. A national cohort study in mainland China demonstrated that ambient PM_2.5_ exposure was associated with an increased risk of PTB in women who conceived through ART treatment (12), which was consistent with our results. Furthermore, we also found that PM_10_ exposure during multiple periods was linked to increased risks of PTB occurrence among the subjects. In line with a study in Xuzhou, China, prenatal exposure to ambient PM_10_ was significantly associated with PTB risk (31). Consistent with a prior study in natural conception (8), our findings not only found significant associations during the specific trimesters and the entire pregnancy, they also showed that the pre-pregnancy periods such as 85 days before oocyte retrieval might be vital windows susceptible to the adverse effects of air pollution in IVF patients. Consistent with our study, a previous study indicated that air pollutants during follicular growth stage was significantly associated with adverse pregnancy outcomes, which provides evidence that air pollutants during the preantral-antral follicle transition stage could influence the quality of women gametes (20, 32). The possible reason might be that the follicle growth is a long process in women, which takes about 85 days for preantral follicles to develop into preovulatory follicles (33). Ambient air pollutants exposure during the follicle growth period could trigger PTBs by directly stimulating inflammation or oxidative stress, which then indirectly activates inflammatory cells (34). Maternal inflammation may alter placental vascular function (35). Moreover, particulate matter can reduce maternal vessel diameters, which may lead to reduced ovarian and uterine blood supply (36). Furthermore, exposure to air pollution may interfere with the delivery of oxygen and nutrients to the placental development during the early stages of pregnancy (37), as these stages represent the beginning of the IVF treatment course. It is plausible that these changes may lead to PTB through inadequate follicle growth or impaired nutrient exchange (38).

In this study, we also observed that ambient NO_2_ exposure was significantly associated with the increased risk of PTB in IVF patients during the 85 days before oocyte retrieval. In contrast to studies reporting increased odds of a low live birth rate or PTB for IVF outcomes with exposure to ambient PM_2.5_ (12, 39), limited estimates were available for the effects of NO_2_ on PTB in patients who underwent IVF. The main source of NO_2_ was derived from vehicle exhaust fumes, particularly in urban areas, and it is widely considered an indicator of traffic-related air pollution (40). Several studies performed in a natural conception population showed that exposure to NO_2_ during the pregnancy or before pregnancy increased the risk of PTB (40, 41), which was similar to our results. The possible mechanisms include oxidative stress, inflammatory reaction, endothelial dysfunction, and endocrine disruption, which may be primary factors of interest to help determine whether these effects could be responsible for PTB (14, 42). While some animal experiments and epidemiological studies have provided no means of distinguishing the NO_2_ effects from those of particulate matter pollutants, especially particles in traffic gaseous emission, as they might well act *via* the similar mechanisms (43). Compared to women who conceive naturally, those who conceived via ART treatment tended to have a higher inflammatory response, as the incidence of female infertility is related to underlying inflammation (14, 42). Furthermore, ART treatment may aggravate inflammation due to repeated ovarian stimulation and embryo transfer (44).

Maternal age plays a fundamental role in achieving successful reproduction and IVF outcomes, as it is inversely linked to oocyte yield and oocyte quality. In the current study, we found that women aged <35 years were more vulnerable to ambient PM_2.5_ than those who were aged ≥ 35 years. One of the possible explanations was that the higher amounts of follicles in young ovarian required more blood supply for nutrition and had more metabolic activity, which could increase the possibility of more air pollutants interacting with the ovarian tissue in young women (20). Lin et al. (45)observed that women aged ≥ 35 years had greater risks of PTB than nulliparous women aged 25–29 years. Specifically, as a woman's age increases, the likelihood of oocytes harboring chromosomal abnormalities and demonstrating cellular dysfunction increases, which results in a decrease in oocyte quality (46). Consequently, the effects of air pollutants exposure on PTB outcomes in IVF patients might be masked by adverse effects related to older reproductive aging. In addition, our results showed that those who underwent two embryos transferred had higher risk than those following single embryo transfer when exposure to ambient particulate matter. Previous studies demonstrated that though the practice of transferring multiple embryos increased the pregnancy rate, whereas the multiple-gestation pregnancies or multiple births enhanced the risks of obstetric complications, PTB and low birth weight (30, 47). Therefore, the adverse effects of transferring double embryos might increase the vulnerability of PTB when exposure to ambient air pollutants.

To our knowledge, this was one of few studies performed in China to investigate the association between time-varying exposure to ambient air pollutants and the risk of PTB in patients who underwent IVF. Firstly, it contributes to filling the knowledge gap of understanding the effects of ambient PM and NO_2_ exposure on PTB occurrence in IVF patients. Secondly, our study helped to identify the potential windows of greater vulnerability to air pollutants exposure, by performing analyses in discrete time not only including the traditional trimesters of pregnancy but also the pre-pregnancy time during the IVF cycle. We highlight that the period 85 days before oocyte retrieval should be given higher priority for immediate for intervention. Finally, our study included the first, fresh embryo(s) transfer cycle performed in our fertility center and adjusted for main confounders, which helped to provide more convincing results. In addition, our stratified analysis enhanced the understanding and emphasized the importance of female reproductive age and number of embryos transferred, these factors which might confound the associations between air pollutants exposure and PTB outcome.

Several limitations in this study should be considered. Firstly, we estimated the average level of ambient air pollutants in subjects' residential locations as a proxy for individual exposures, the measurement error in exposure tends to cause bias toward a null (48), which might underestimate the association. While, the results are not likely to be exaggerated. We also lacked information on the paternal exposure in the study, which might play a potential role in the embryo development in IVF treatment. Secondly, our study only explored the associations in patients who underwent fresh embryo transfer cycle, due to a lack of information about frozen–thawed embryo transfer. Thirdly, limited by data, the information regarding social-economic status, ambient ultrafine particles concentration, and indoor sources of pollution, such as type of heating or cooking fuel in participants' homes were not available; which might introduce potential additional confounders in our study. Finally, the sample size was relatively limited due to the scale of the center, and the findings should be interpreted with caution. Consequently, a prospective cohort study with a larger population should be performed to obtain more reliable results.

## Conclusions

Our study suggested that ambient air pollutants, such as PM_2.5_, PM_10_, and NO_2_ exposure were significantly associated with an increased risk of PTB incidence in patients who underwent IVF in Hangzhou, China. This association was more pronounced in women <35 years of age and those who undergoing two embryos transferred. Furthermore, the specific effects of air pollutants exposure, particularly during the time windows from 85 days before oocyte retrieval, gonadotropin start to oocyte retrieval, first trimester of pregnancy, and the entire IVF pregnancy, exhibited significant association with PTB risk. Our study provided epidemiological evidence for adverse effects of air pollution on PTB risk in IVF patients. Future prospective studies are needed to explore the possible mechanisms of ambient air pollution on PTB outcome, and further confirm our findings.

## Data Availability Statement

The raw data supporting the conclusions of this article will be made available by the authors, without undue reservation.

## Ethics Statement

The studies involving human participants were reviewed and approved by the Medical Ethics Committee of the Hangzhou Women's Hospital. The Ethics Committee waived the requirement of written informed consent for participation.

## Author Contributions

WS and CJ: study concept and design. WS, MJ, and XF: acquisition of data. WS, MJ, LK, and CJ: analysis and interpretation of data. WS: statistical analysis. WS and MJ: drafting of the manuscript. WS, LK, TZ, QY, ZW, SX, and CJ: edit and critical revision of the manuscript. XF and CJ: study supervision. All authors contributed to the article and approved the submitted version.

## Funding

This study was supported by the Medical and Health Science and Technology Project of Zhejiang Province (No. 2020KY764).

## Conflict of Interest

The authors declare that the research was conducted in the absence of any commercial or financial relationships that could be construed as a potential conflict of interest.

## Publisher's Note

All claims expressed in this article are solely those of the authors and do not necessarily represent those of their affiliated organizations, or those of the publisher, the editors and the reviewers. Any product that may be evaluated in this article, or claim that may be made by its manufacturer, is not guaranteed or endorsed by the publisher.
