# Supplementary material for: Association Between Ambient Air Pollutants Exposure and Preterm Birth in Women Who Underwent in vitro Fertilization: A Retrospective Cohort Study From Hangzhou, China
Source: Front Med (Lausanne). 2021 Dec 13;8:785600. doi: 10.3389/fmed.2021.785600 (PMC8710591; doi:10.3389/fmed.2021.785600)
Supplement: Supplementary file 1 [file Data_Sheet_1.doc]

**Supporting information**

**Association between ambient air pollutants exposure and preterm birth in women who underwent in vitro fertilization: A retrospective cohort study from Hangzhou, China**

**Contents**

**Table S1.** Spearman's rank correlations between paired air pollutants in different time periods.

**Table S2.** Spearman's rank correlations of air pollutants over time during different periods of IVF pregnancy among the subjects.

**Table S3.** Adjusted hazard ratios and 95%confidence intervals of preterm birth associated with per interquartile range increment of air pollutants during IVF pregnancy (Period I) in both the single-pollutant and two-pollutant models ^a^

**Figure S1.** Summary statistics of the ambient average exposure levels of six air pollutants in different time periods during the IVF pregnancy among the subjects.

**Figure S2.** Comparsion of the association between time-varying air pollutants and incident PTB during different exposure periods and the effect by further adjusting for baseline concentration in period A.

**
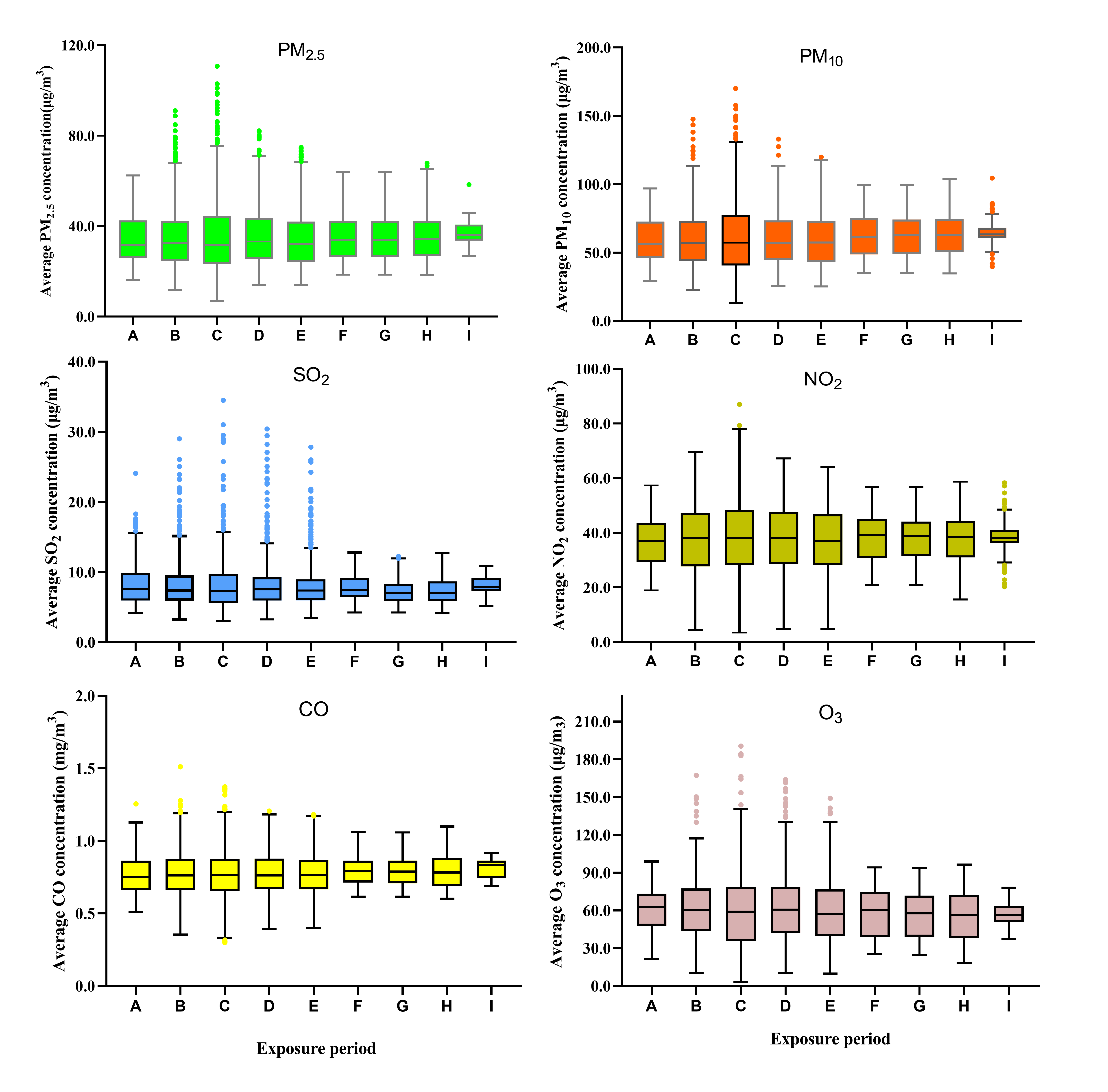
**

**Figure S1.** Summary statistics of the ambient average exposure levels of six air pollutants in different time periods during the IVF pregnancy among the subjects.

**Table S1.** Spearman's rank correlations between paired air pollutants in different exposure periods.

|  |  | PM_2.5_ | PM_10_ | NO_2_ | SO_2_ | CO | O_3_ |
| --- | --- | --- | --- | --- | --- | --- | --- |
| Period A | PM_10_ | 0.957** |  |  |  |  |  |
|  | NO_2_ | 0.859** | 0.917** |  |  |  |  |
|  | SO_2_ | 0.689* | 0.608* | 0.479* |  |  |  |
|  | CO | 0.837** | 0.818** | 0.761** | 0.685** |  |  |
|  | O_3_ | -0.600** | -0.581* | -0.726** | -0.201** | -0.578** | 1.00 |
|  |  |  |  |  |  |  |  |
| Period B | PM_10_ | 0.944** |  |  |  |  |  |
|  | NO_2_ | 0.833** | 0.878** |  |  |  |  |
|  | SO_2_ | 0.673** | 0.612* | 0.478** |  |  |  |
|  | CO | 0.793** | 0.708** | 0.716** | 0.582* |  |  |
|  | O_3_ | -0.397* | -0.350* | -0.524** | -0.121* | -0.526** | 1.00 |
|  |  |  |  |  |  |  |  |
| Period C | PM_10_ | 0.934** |  |  |  |  |  |
|  | NO_2_ | 0.782** | 0.800** |  |  |  |  |
|  | SO_2_ | 0.664** | 0.619** | 0.500** |  |  |  |
|  | CO | 0.706** | 0.603* | 0.666** | 0.619** |  |  |
|  | O_3_ | -0.234* | -0.164* | -0.532** | -0.075* | -0.479** | 1.00 |
|  |  |  |  |  |  |  |  |
| Period D | PM_10_ | 0.889** |  |  |  |  |  |
|  | NO_2_ | 0.758** | 0.832** |  |  |  |  |
|  | SO_2_ | 0.643** | 0.604** | 0.458* |  |  |  |
|  | CO | 0.783** | 0.709** | 0.688** | 0.581** |  |  |
|  | O_3_ | -0.451** | -0.400** | -0.661** | -0.142** | -0.529** | 1.00 |
|  |  |  |  |  |  |  |  |
| Period E | PM_10_ | 0.948** |  |  |  |  |  |
|  | NO_2_ | 0.782** | 0.855** |  |  |  |  |
|  | SO_2_ | 0.635** | 0.593** | 0.652** |  |  |  |
|  | CO | 0.803** | 0.735** | 0.694** | 0.562** |  |  |
|  | O_3_ | -0.443* | -0.412* | -0.531** | -0.115* | -0.535* | 1.00 |
|  |  |  |  |  |  |  |  |
| Period F | PM_10_ | 0.967** |  |  |  |  |  |
|  | NO_2_ | 0.837** | 0.910** |  |  |  |  |
|  | SO_2_ | 0.683** | 0.615** | 0.643* |  |  |  |
|  | CO | 0.882** | 0.827** | 0.729** | 0.511* |  |  |
|  | O_3_ | -0.624** | -0.565* | -0.567* | -0.277* | -0.534* | 1.00 |
|  |  |  |  |  |  |  |  |
| Period G | PM_10_ | 0.956** |  |  |  |  |  |
|  | NO_2_ | 0.834** | 0.899** |  |  |  |  |
|  | SO_2_ | 0.692** | 0.603** | 0.626* |  |  |  |
|  | CO | 0.838** | 0.740** | 0.685** | 0.520** |  |  |
|  | O_3_ | -0.652* | -0.589* | -0.485* | -0.182 | -0.453* | 1.00 |
|  |  |  |  |  |  |  |  |
| Period H | PM_10_ | 0.969** |  |  |  |  |  |
|  | NO_2_ | 0.852** | 0.918** |  |  |  |  |
|  | SO_2_ | 0.508** | 0.694** | 0.673* |  |  |  |
|  | CO | 0.880** | 0.825** | 0.760** | 0.633** |  |  |
|  | O_3_ | 0.755** | -0.522* | -0.492* | -0.171* | -0.520* | 1.00 |
|  |  |  |  |  |  |  |  |
| Period I | PM_10_ | 0.970** |  |  |  |  |  |
|  | NO_2_ | 0.722** | 0.726** |  |  |  |  |
|  | SO_2_ | 0.912** | 0.876** | 0.524** |  |  |  |
|  | CO | 0.898** | 0.893** | 0.623** | 0.580** |  |  |
|  | O_3_ | -0.459* | -0.424* | -0.368* | -0.352* | -0.233* | 1.00 |

**P* < 0.05, ** *P* < 0.01

**Table S2.** Spearman's rank correlations of air pollutants over time during different periods of IVF pregnancy among the subjects.

| **PM_2.5_** | Period A | Period B | Period C | Period D | Period E | Period F | Period G | Period H |
| --- | --- | --- | --- | --- | --- | --- | --- | --- |
| Period B | 0.751** |  |  |  |  |  |  |  |
| Period C | 0.567** | 0.741** |  |  |  |  |  |  |
| Period D | 0.580** | 0.732** | 0.675** |  |  |  |  |  |
| Period E | 0.313* | 0.624** | 0.557** | 0.740** |  |  |  |  |
| Period F | 0.325** | 0.669** | 0.624** | 0.824** | 0.903** |  |  |  |
| Period G | 0.588** | 0.410** | 0.287* | 0.302* | 0.312** | 0.235* |  |  |
| Period H | 0.278* | 0.368** | 0.351* | 0.571** | 0.622** | 0.641** | 0.139* |  |
| Period I | 0.368** | 0.286* | 0.259* | 0.185* | 0.191* | 0.272* | 0.178* | 0.324** |
|  |  |  |  |  |  |  |  |  |
| **PM_10_** | Period A | Period B | Period C | Period D | Period E | Period F | Period G | Period H |
| Period B | 0.680** |  |  |  |  |  |  |  |
| Period C | 0.490** | 0.679** |  |  |  |  |  |  |
| Period D | 0.489** | 0.678** | 0.650** |  |  |  |  |  |
| Period E | 0.227** | 0.542** | 0.509** | 0.744** |  |  |  |  |
| Period F | 0.178* | 0.575** | 0.547** | 0.778** | 0.872** |  |  |  |
| Period G | 0.624** | 0.454** | 0.358** | 0.346** | 0.489** | 0.135* |  |  |
| Period H | 0.187* | 0.353** | 0.306** | 0.459** | 0.524** | 0.616** | 0.122* |  |
| Period I | 0.243** | 0.382** | 0.266** | 0.226** | 0.265** | 0.219** | 0.141* | 0.259* |
|  |  |  |  |  |  |  |  |  |
| **SO_2_** | Period A | Period B | Period C | Period D | Period E | Period F | Period G | Period H |
| Period B | 0.784** |  |  |  |  |  |  |  |
| Period C | 0.626** | 0.795** |  |  |  |  |  |  |
| Period D | 0.620** | 0.769** | 0.798** |  |  |  |  |  |
| Period E | 0.171* | 0.300** | 0.342** | 0.404** |  |  |  |  |
| Period F | 0.484** | 0.577** | 0.552** | 0.650** | 0.297** |  |  |  |
| Period G | 0.341** | 0.312** | 0.293** | 0.286** | 0.344** | 0.369** |  |  |
| Period H | 0.484** | 0.358** | 0.316** | 0.275** | 0.247** | 0.227** | 0.385** |  |
| Period I | 0.638** | 0.536** | 0.499** | 0.494** | 0.272** | 0.456** | 0.594** | 0.786** |
|  |  |  |  |  |  |  |  |  |
| **NO_2_** | Period A | Period B | Period C | Period D | Period E | Period F | Period G | Period H |
| Period B | 0.713** |  |  |  |  |  |  |  |
| Period C | 0.587** | 0.823** |  |  |  |  |  |  |
| Period D | 0.472** | 0.782** | 0.801** |  |  |  |  |  |
| Period E | 0.241** | 0.561** | 0.619** | 0.794** |  |  |  |  |
| Period F | 0.182* | 0.570** | 0.633** | 0.788** | 0.890** |  |  |  |
| Period G | 0.483** | 0.463** | 0.445** | 0.401** | 0.248** | 0.220** |  |  |
| Period H | 0.202** | 0.345** | 0.374** | 0.412** | 0.339** | 0.439** | 0.267** |  |
| Period I | 0.306** | 0.247** | 0.209** | 0.212** | 0.166* | 0.207** | 0.107* | 0.301** |
|  |  |  |  |  |  |  |  |  |
| **CO** | Period A | Period B | Period C | Period D | Period E | Period F | Period G | Period H |
| Period B | 0.806** |  |  |  |  |  |  |  |
| Period C | 0.653** | 0.796** |  |  |  |  |  |  |
| Period D | 0.677** | 0.804** | 0.753** |  |  |  |  |  |
| Period E | 0.511** | 0.674** | 0.622** | 0.798** |  |  |  |  |
| Period F | 0.417** | 0.647** | 0.593** | 0.759** | 0.826** |  |  |  |
| Period G | 0.194* | 0.286** | 0.247** | 0.235** | 0.282** | 0.204** |  |  |
| Period H | 0.274** | 0.365** | 0.267** | 0.357** | 0.270** | 0.321** | 0.262** |  |
| Period I | 0.428** | 0.372** | 0.347** | 0.329** | 0.350** | 0.442** | 0.601** | 0.565** |
|  |  |  |  |  |  |  |  |  |
| **O_3_** | Period A | Period B | Period C | Period D | Period E | Period F | Period G | Period H |
| Period B | 0.653** |  |  |  |  |  |  |  |
| Period C | 0.477** | 0.776** |  |  |  |  |  |  |
| Period D | 0.421** | 0.707** | 0.754** |  |  |  |  |  |
| Period E | 0.279** | 0.523** | 0.576** | 0.758** |  |  |  |  |
| Period F | 0.233** | 0.591** | 0.616** | 0.776** | 0.867** |  |  |  |
| Period G | 0.724** | 0.333** | 0.235** | 0.203** | 0.207** | 0.348** |  |  |
| Period H | 0.302** | 0.584** | 0.587** | 0.640** | 0.658** | 0.760** | 0.250* |  |
| Period I | 0.344** | 0.280** | 0.252** | 0.235** | 0.338** | 0.416** | 0.311** | 0.218* |

**P* < 0.05, ** *P* < 0.01

**Table S3.** Adjusted hazard ratios and 95%confidence intervals of preterm birth associated with per interquartile range increment of air pollutants during IVF pregnancy (Period I) in both the single-pollutant and two-pollutant models a

|  | Single-pollutant model | Two-pollutant model | | | | | |
| --- | --- | --- | --- | --- | --- | --- | --- |
|  |  | Adjusted for PM_2.5_ | Adjusted for PM_10_ | Adjusted for SO_2_ | Adjusted for NO_2_ | Adjusted for CO | Adjusted for O_3_ |
| PM_2.5_ | **1.07 (1.01-1.14)** | **—** | **1.12 (1.01-1.23)*** | **1.07 (1.03-1.18)*** | **1.08 (1.02-1.15)*** | 1.04 (0.98-1.10) | **1.09 (1.04-1.21)*** |
| PM_10_ | 1.08 (0.95-1.24) | 1.06 (0.92-1.19) | **—** | 1.08 (0.92-1.23) | 1.04 (0.92-1.17) | 1.05 (0.94-1.21) | 1.11 (0.96-1.28) |
| SO_2_ | 0.86 (0.65-1.15) | 0.88 (0.66-1.15) | 0.88 (0.67-1.18) | **—** | 0.89 (0.70-1.14) | 0.84 (0.62-1.19) | 0.83 (0.62-1.11) |
| NO_2_ | 1.02 (0.89-1.17) | 1.07 (0.88-1.32) | 1.12 (0.92-1.37) | 1.05 (0.90-1.22) | **—** | 1.04 (0.86-1.25) | 0.97 (0.83-1.13) |
| CO | 1.03 (0.86-1.26) | 1.00 (0.82-1.21) | 1.02 (0.84-1.22) | 1.05 (0.87-1.26) | 1.07 (0.89-1.29) | **—** | 1.02 (0.83-1.20) |
| O_3_ | 1.06 (0.76-1.47) | 0.98 (0.90-1.05) | 1.01 (0.92-1.13) | 0.99 (0.91-1.06) | 0.99 (0.89-1.04) | 1.00 (0.90-1.09) | **—** |

a: Model adjusted for maternal age, maternal pre-pregnancy BMI, educational level, employment status, cigarette smoking exposure, residential locations, mean ambient temperature and relative humidity.

*P < 0.05


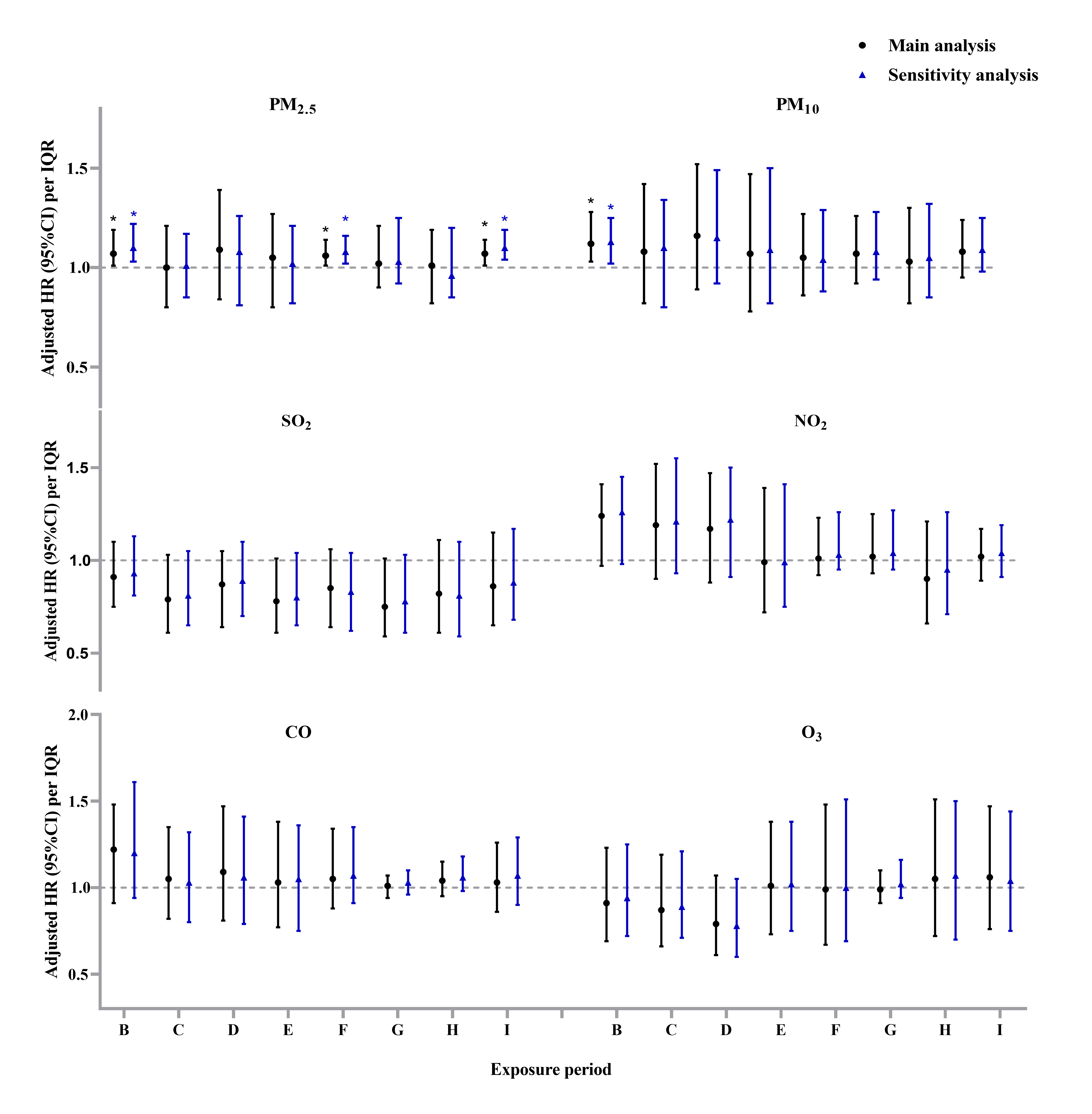


**Figure S2.** Comparsion of the association between time-varying air pollutants and incident PTB during different exposure periods and the effect by further adjusting for baseline concentration in period A.

**P* <0.05
